# Genome Wide Association Study to predict severe asthma exacerbations in children using random forests classifiers

**DOI:** 10.1186/1471-2350-12-90

**Published:** 2011-06-30

**Authors:** Mousheng Xu, Kelan G Tantisira, Ann Wu, Augusto A Litonjua, Jen-hwa Chu, Blanca E Himes, Amy Damask, Scott T Weiss

**Affiliations:** 1 Channing Laboratory, Brigham and Women's Hospital, Harvard Medical School, Boston, MA, USA; 2 Bioinformatics Program, Boston University, Boston, MA, USA; 3 Division of Pulmonary and Critical Care Medicine, Brigham and Women's Hospital, Harvard Medical School, Boston, MA, USA; 4 Novartis, Cambridge, MA, USA

## Abstract

**Background:**

Personalized health-care promises tailored health-care solutions to individual patients based on their genetic background and/or environmental exposure history. To date, disease prediction has been based on a few environmental factors and/or single nucleotide polymorphisms (SNPs), while complex diseases are usually affected by many genetic and environmental factors with each factor contributing a small portion to the outcome. We hypothesized that the use of random forests classifiers to select SNPs would result in an improved predictive model of asthma exacerbations. We tested this hypothesis in a population of childhood asthmatics.

**Methods:**

In this study, using emergency room visits or hospitalizations as the definition of a severe asthma exacerbation, we first identified a list of top Genome Wide Association Study (GWAS) SNPs ranked by Random Forests (RF) importance score for the CAMP (Childhood Asthma Management Program) population of 127 exacerbation cases and 290 non-exacerbation controls. We predict severe asthma exacerbations using the top 10 to 320 SNPs together with age, sex, pre-bronchodilator FEV1 percentage predicted, and treatment group.

**Results:**

Testing in an independent set of the CAMP population shows that severe asthma exacerbations can be predicted with an Area Under the Curve (AUC) = 0.66 with 160-320 SNPs in comparison to an AUC score of 0.57 with 10 SNPs. Using the clinical traits alone yielded AUC score of 0.54, suggesting the phenotype is affected by genetic as well as environmental factors.

**Conclusions:**

Our study shows that a random forests algorithm can effectively extract and use the information contained in a small number of samples. Random forests, and other machine learning tools, can be used with GWAS studies to integrate large numbers of predictors simultaneously.

## Background

Personalized medicine, the ability to predict an individual's predisposition to disease and response to therapy with genetic and phenotypic characteristics, promises to deliver more efficient health outcomes [1-4]. As a field, personalized medicine faces multiple issues when trying to predict complex diseases such as cardiovascular diseases, cancer, and asthma. This is largely due to the fact that no single genotypic or phenotypic characteristic can explain more than a small portion of any complex disease. Instead, complex diseases are influenced by multiple genetic factors and environmental exposures. For instance, the height of a person is considered to be strongly heritable, but the top 20 single nucleotide polymorphisms (SNPs) chosen by p value, explain only ~2-3% of the variability in adult height [5]. In addition to the multitude of factors influencing complex traits, the genetic and environmental factors interact with each other adding to the complexity.

To integrate multiple genetic and environmental predictors into modeling, conventional statistical methods and some data mining algorithms such as an artificial neural network (ANN) can be easily over-fit typically due to a small sample size in relation to the number of potential SNPs or predictors. Nevertheless, data mining methods are available to handle this type of data that are more resistant to over-fitting. Random Forests (RF) [6,7] are a classification algorithm that is composed of a set of random decision trees, with each tree making a decision and voting for the final prediction outcome. Being able to generate a highly accurate classifier with many (even relatively weak) predictors without over-fitting [6,7], Random Forests would appear to be an ideal approach to the integration of hundreds of SNPs plus clinical traits needed to predict complex clinical phenotypes. An added benefit of Random Forests is that the decision trees naturally handle interactions among input variables.

Asthma is a complex disease known to be influenced by both genetic and environmental factors [8-16]. 26.7 million or about 9.7% of the population in the United States have had asthma during their lifetime [17]. In the year 2000, asthma exacerbations resulted in 1,499 deaths, 1.1 million hospital days, and $2.9 billion in direct expenditures in the United States [18]. The ability to predict severe asthma exacerbations would therefore have direct prognostic significance and might form the basis for the development of novel therapeutic interventions. Severe asthma exacerbations have been associated with several clinical factors including the forced expiratory volume in one second as a percent of predicted (FEV_1_%), oral corticosteroid usage [9,19], age [20], and sex [21]. However, these factors by themselves are limited in their ability to successfully predict severe asthma exacerbations [21,22]. To explore the potential power of a multi-SNP model as incorporated into RF together with clinical relevant risk factors to effectively predict complex diseases, we applied this algorithm to the prediction of exacerbations in a population of childhood asthmatics participating in the Childhood Asthma Management Program (CAMP).

## Methods

### Study Population

CAMP was a multicenter, randomized, double-blinded clinical trial testing the safety and efficacy of inhaled budesonide vs. nedocromil vs. placebo over a mean of 4.3 years. Trial design, methodology, and primary clinical outcome have been previously published (The Childhood Asthma Management Program Research Group 1999; The   > Childhood Asthma Management Program Research Group 2000). Entry criteria included asthma symptoms and/or medication use for ≥ 6 months in the previous year and airway responsiveness with a provocative concentration dose (PC_20_) of methacholine ≤ 12.5 mg/ml. 1,041 children with mild-moderate asthma were enrolled.

At baseline, data regarding demographics; home environment characteristics; asthma symptoms, severity, and treatment; allergy history; and relevant family history were collected. Each patient's parent or guardian signed a consent statement, with each child providing assent. IRB approval was obtained for all participating CAMP centers and the data coordinating center. A 6 week run-in period which included therapy limited to as needed albuterol preceded randomization. Visits occurred at randomization, at two and four months after randomization, and every four months thereafter. During these visits, an interval asthma history was obtained, including specific questions related to health care utilization related to asthma.

The CAMP Genetics Ancillary Study was approved by each individual study center's Internal Review Board, and informed consent/assent was obtained from all participants and their parents.

### Primary outcome

The occurrence of either an emergency room visit or a hospitalization for asthma symptoms at any time during the clinical trial period was used to define a severe asthma exacerbation.

### Clinical covariates

Age, sex, pre-bronchodilator FEV_1_%, and treatment group are known to be associated with asthma exacerbations and were included as clinical traits in our models. Values for each predictor were those obtained at the CAMP randomization visit. Age and pre-bronchodilator FEV_1_%, are coded as numeric variables; sex is coded as 1 for male, 2 for female; treatment group is coded as 1, 2, 3 for three different treatments.

### GWAS data

Of the CAMP participants, 422 Caucasian parent-child trios were genotyped using the Infinium II HumanHap550v3 Genotyping BeadChip (Illumina, San Diego, CA), 164 Caucasian non-trio cohort children were subsequently genotyped using the Human660W-Quad BeadChip. 5 of the 422 trios had an excess of missing genotypes and were removed from this study, and thus 417 trio children were actually used in this study. Over 500,000 SNPs were successfully genotyped in the CAMP trios, with a reproducibility of > 99.99%. Reproducibility is based on 4 samples that were each genotyped 15 times in the experiment. Genotype quality has been validated using the Mendel option of PLINK v0.99r http://pngu.mgh.harvard.edu/purcell/plink/[23], verifying allele calls against RefSeq to ensure correct orientation, and testing for extreme departures from Hardy Weinburg equilbrium in the parents.

### Selection of SNPs

Focusing on the trio probands as our initial test population, we used RF importance scores to rank and select SNPs in two steps. At each step, we used SNPs as predictors to predict asthma exacerbations with RF, and obtained the RF importance score of each of the SNPs. At the first step, we computed RF importance scores for all SNPs genome-wide, 4,000 at a time, in chromosomal order. At the second step, we ranked all SNPs based on their RF importance scores, selected the top 4,000 SNPs, and reran RF with these selected SNPs to re-rank them.

### Prediction model building with RF

The 417 Caucasian trio samples (Stage 1 samples) were genotyped before the 164 cohort samples (Stage 2 samples), and were used to build and train the RF models to predict asthma exacerbations. The R package randomForest version 4.5-25 originally written in Fortran by Leo Breiman and Adele Cutler and ported to R by Andy Liaw and Matthew Wiener http://cran.r-project.org/web/packages/randomForest/index.html was used to build RF models in this study. The RF predicted score is the percentage of trees voting for "yes". During this step and the steps described in "**Selection of SNPs**" above, RF parameter "ntree" (number of trees to grow) were set to be 1,500 - a relatively large number to ensure stable prediction results, and all other parameters, including mtry, were set to use the default values.

### Prediction Performance controls

In order to assess the performance of the RF classifier built with the selected clinical traits and SNPs as predictors, two types of controls were used in this study. One type of control is called a **permutation control**, the other a **random SNP control**. The permutation control permuted the response variable (any severe exacerbation) among samples while retaining the association of the predictors with the samples; the random SNP control randomly selected SNPs used in the Genome-Wide Association Study (GWAS) regardless of whether they are associated with the phenotype or not, and used the equivalent number of random SNPs to build predictive models. Both controls were iterated 10 times.

### Testing in an independent population

After the RF models were built with the Stage 1 samples, additional samples were genotyped and used as the Stage 2 population for testing. The clinical traits and SNPs of the Stage 2 samples were used to predict the asthma exacerbations with these RF models. Because RF does not allow missing values for prediction, missing alleles were imputed by randomly selecting a genotype based on the observed genotype frequency distribution among controls.

### ROC curve and AUC computation

Receiver Operating Characteristic curve (ROC curve) and the Area Under the ROC Curve (AUC) were computed using the R package ROCR developed by Sing et al (Sing 2005), which is downloadable from http://rocr.bioinf.mpi-sb.mpg.de/. We used the AUC as our primary indicator of predictive success [24,25]. The computation of the p-value for an AUC to be different from that would be obtained by chance is described in [24,26].

## Results

### Sample characteristics

The clinical characteristics of both our training (trios) and test (probands) are shown in Table 1. The Stage 1 samples are 417 CAMP asthmatic family-trio children genotyped and used in this study. 127 (30%) of them experienced at least one severe asthma exacerbations during the four year follow-up period as indicated by an emergency room visit or hospitalization (Table 1). Children with severe exacerbations were more likely to be male, have lower pre-bronchodilator FEV_1_%, and be untreated with drugs. The Stage 2 samples are 164 cohort children, who are independent of the Stage 1 samples. A similar percentage of the Stage 2 children experienced exacerbations in the follow-up period when compared with Stage 1 samples. Two of the four clinical traits (gender and treatment group) in the study are different in the Stage 2 samples from the Stage 1 samples. The clinical traits are used as covariates for the outcome prediction.

**Table 1:** Sample Characteristics (All Subjects Are Caucasian)

	Training Population N = 417		Testing Population N = 164	
	**Exac.**	**Non-exac.**	**Exac.**	**Non-exac.**

Subjects	127 (30%)	290 (70%)	50 (30%)	114 (70%)
Age (mean ± s.d.)	8.41 ± 2.07	8.89 ± 2.07	8.54 ± 2.28	9.45 ± 2.19
Male	69%	61%	46%	53%
FEV1% (mean ± s.d.)	92.9 ± 15.7	93.5 ± 13.2	95.4 ± 17.1	95.4 ± 13.5
Treatment				
Budesonide	20%	31%	36%	32%
Nedocromil	28%	29%	30%	32%
Placebo	52%	39%	34%	36%

### The importance score landscape of SNPs genome-wide

**Figure 1:**
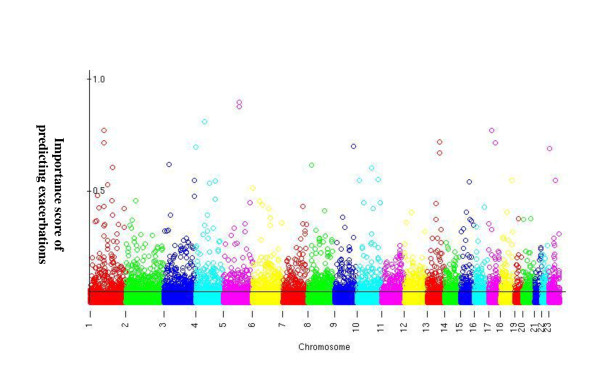
**The "manhattan plot" of RF importance scores of all the 550k SNPs**. X-axis: the SNPs in chromosomal order; Y-axis: the RF importance scores. The black demarcation separates the top 4k SNPs from the rest.

RF importance score measures the relative contribution of a predictor to the prediction. The importance score of each of the SNPs is plotted in Figure 1 in chromosomal order. The demarcation separates the top 4k SNPs with the highest importance scores from the rest. This plot is similar to the "Manhattan plot" seen in GWAS analysis except the y-axis is the RF importance score instead of the -log(p).

### Prediction of severe asthma exacerbations

**Figure 2:**
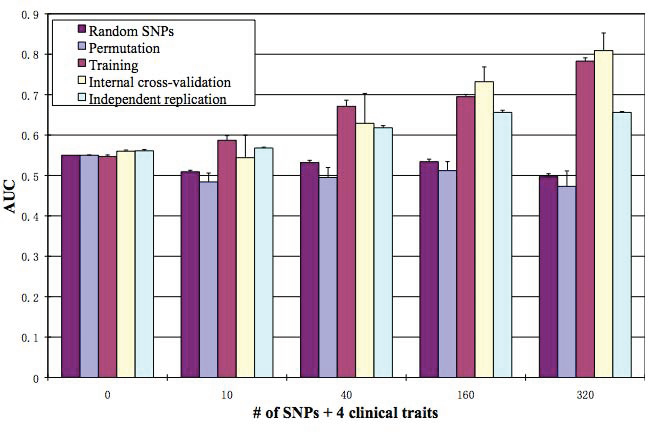
**Comparison of performance of predicting severe asthma exacerbation with different methods**. Y-axis: AUC; X-axis: the number of SNPs used in a model. "Random SNPs": SNPs are chosen randomly from all SNPs and used as input variables to predict asthma exacerbations, and this process has been iterated 10 times [see Methods for details]; "Permuted": asthma exacerbation is permuted across samples while clinical traits and SNPs are kept with the samples, and this process has been iterated 10 times [see Methods for details]; "Training": the AUC of the model trained and built with all the Stage 1 samples predicting on the same samples; "Internal cross-validation": the AUC of the model built with 90% of the randomly selected Stage 1 samples predicting on the rest (10%) of the Stage 1 samples; "Independent replication": the AUC of the model built with all the Stage 1 samples predicting on all the Stage 2 samples.

**Figure 3:**
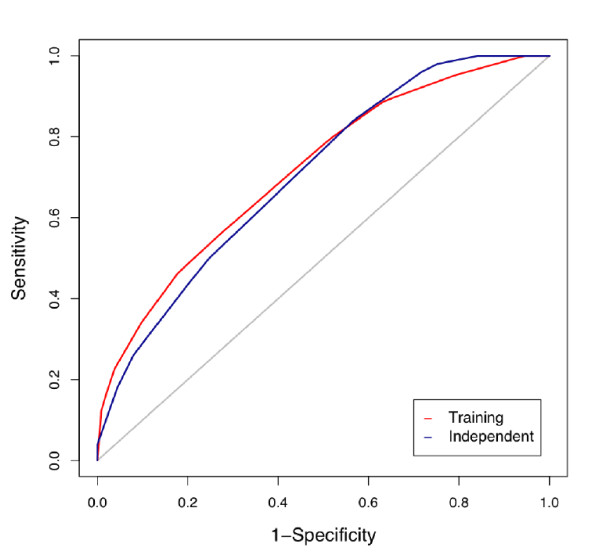
**ROC curves using clinical attributes plus 160 SNPs as predictors**. The red curve is obtained for the training of the Stage 1 samples, the blue curve is for the testing of the Stage 2 samples, the grey diagonal line is a theoretical curve representing random guess. Both the red and the blue curves are higher than the grey line, indicating better than random prediction; and they are similar to each other, suggesting the true predictability of the RF model. The p-value for the independent testing AUC to be different from 0.5 is 0.000266.

Using clinical traits age, sex, pre-bronchodilator FEV_1_%, and treatment group, plus different numbers of SNPs selected based on RF importance score (see Methods) as predictors, RF predicted severe asthma exacerbations with varying degrees of success. With just the 4 clinical attributes as predictors, the predictive model had an externally replicated AUC of about 0.56 (Figure 2). Since, an AUC of 0.5 indicates prediction equivalent to chance, clinical predictors alone had weak predictability. The addition of the 10 SNPs with the highest RF importance score for exacerbations increased the AUC to 0.57. The addition of SNPs continued to increase the predictability of asthma exacerbations, with an independently replicated AUC of 0.62, 0.66, and 0.66 for 40, 160 and 320 SNPs, respectively. The ROC curves for prediction using 160 SNPs in the training and independent populations are shown in Figure 3. The p-value for the independent replication AUC 0.66 to be different from 0.5 by random guess is 0.000266. Starting at 160 SNPs the AUC reached 0.66 and began to plateau with additional SNPs. The top 160 SNPs together with the closest genes are listed in Additional File 1, Table S1.

### Model validation

To evaluate the RF models, we performed permutation and random SNP controls and used an independent replication population (Figure 2). *Permutation control*: after permuting exacerbations labels among samples, RF models were built and tested for predictability in terms of AUC. For all RF models built with permuted data, the AUC scores were about 0.5, suggesting the true predictability of RF models built with original data. *Random SNP control*: random SNPs showed AUC scores slightly higher than 0.5 (data not shown). The evidence for weak predictability in the Random SNP control models likely comes from the clinical traits used in the models rather than the random SNPs. These results indicate that the RF selected SNPs contain information about exacerbation, while the random SNPs do not.

## Discussion

One of the biggest challenges of complex trait prediction is the lack of statistical power that is the direct result of small effects of many causal factors and relatively small sample sizes. These problems are exacerbated when consideration is given to the potential for interaction among the causal factors to interact with one another. We have shown that RF modeling can produce accurate results using hundreds of SNPs obtained from a relatively small study (131 cases, 291 controls). With only 417 subjects, using 160 SNPs, we are able to generate a good predictive model for childhood asthma exacerbations, with a > 0.66 AUC and about 0.66 sensitivity and 0.6 specificity (Figures 2, 3). Depending on the portion of the ROC curve that is used, this can equate to a positive predictive value (PPV) of 0.81 and a negative predictive value (NPV) of 0.74 with proportion of exacerbators = 0.3 as shown in Table 1 and choosing a scoring threshold corresponding to sensitivity = 0.2 and specificity = 0.95, allowing for reasonable prediction of asthma exacerbations. The permutation control, random SNP control, and independent replication results all support the validity and robustness of the random forest predictive model. The ROC curve obtained for the model training using the Stage 2 (independent replication) samples is very similar to that obtained for the Stage 1 (training) samples (Figure 3), and the p-value for the independent replication AUC is < 0.05, indicating reproducibility of the predictive accuracy in the model using 160 SNPs.

The 160 SNPs are in or near to 140 genes (Additional File 1, Table S1). Among the top 160 SNPs, one SNP (rs10496476) is located within the intron of gene DPP10, which has been shown to be associated with asthma in multiple populations, based on a recent review [27]. All other genes are not on the list of replicated asthma genes reported by [27], suggesting most SNPs and genes identified by our RF method are novel. A couple of factors may contribute to the discovery of new SNPs and genes: 1) RF evaluates individual SNPs in the context of interactions. This is different from conventional statistical methods such as logistic regression, applied in GWAS which searches SNPs one by one without consideration of SNP-SNP interactions; 2) asthma exacerbations are related to but different phenotypes from asthma diagnosis.

Our study highlights an innovative way of integrating a large number of individually weak predictors to effectively build a reasonable predictive model for asthma exacerbations. Given that complex trait studies so far have used only up to a dozen predictors (i.e. an order of magnitude fewer than what we use here) with limited consideration of interaction and have generated relatively poor predictability, our approach of employing RF with hundreds of predictors with a relatively small sample size gives hope for additional improvement in complex trait prediction using a variety of machine learning approaches. Talmud, et al, studied 20 SNPs derived from genome-wide association studies of type 2 diabetes susceptibility in a population of 5,535 subjects followed for 10 years [28]. They noted that clinical factors outperformed genetic markers in the prediction of incident diabetes and that the addition of the SNPs produced minimal improvement in risk estimation based only on clinical variables. In contrast to this study, which focused on the additive effects of 20 SNPs, our RF model simultaneously accounts for both additive and interactive effects using 160 SNPs to more effectively predict asthma exacerbations compared with clinical factors alone.

Most complex traits such as adult height, cardiovascular diseases, cancer, diabetes, autism, and asthma etc. are likely to be encoded by a large number of both genetic and environmental factors [5,29-32]. Asthma exacerbations, as shown in this study, is associated with at least several hundred genetic markers and environmental factors. The top 10 SNPs has AUC score 0.57, showing marginal predictability. But with 160 SNPs, the AUC of the RF model approached 0.66. Our study suggests that in order to get good prediction of a complex trait, methods capable of integrating hundreds of predictors, such as machine learning approaches, like random forests, will be valuable.

Asthma exacerbations have historically been difficult to predict. Several clinical models have been designed to try to enhance the ability to predict exacerbations [21,33-36]. Most studies [21,33,36] attempted to isolate predictive clinical variables individually without accounting for interaction using odds ratio or regression analysis. The results of these studies are reported as odds ratios or as p-values for individual factors, and cannot be directly compared with ours using AUC.

There have been several publications that have evaluated the use of a clinical classification tree in the development of a prognostic model for asthma exacerbations [34]. One study evaluated six clinical variables including prior year hospitalization, the classification tree method was able to achieve - without independent replication - 94% sensitivity and 68% specificity, better than logistic regression (87% sensitivity and 48% specificity) or an additive risk model (46% sensitivity and 93% specificity), suggesting the value of accounting for interactions among predictive variables. A recent study [35] reported 66.8% sensitivity and 85.8% specificity (with no independent validation testing) on childhood asthma exacerbations (defined as rescue oral corticosteroid use, an unscheduled visit to a physician or emergency room, or hospitalization) prediction with daytime cough, daytime wheeze, and β2-agonist use at night 1 day before the exacerbations as predictors. However, none of the clinical models developed to date have been independently validated. In our model, we successfully used both internal (e.g. permutation) controls as well as external replication in an independent subset of subjects to demonstrate predictive power of our model. While the independent subset of subjects were derived from the same source population, there were some differences in baseline characteristics between the two samples (Table 1), further supporting the generalizability of our model. RF also uses a classification tree, but with a difference - it uses many classification trees (1500 trees in our models), not just one, and it can handle a greater number of input variables without over-fitting.

A critical issue for complex disease prediction is the difficulty of extending the predictive power of a model obtained from one population to an independent population. None of the studies mentioned in the proceeding paragraph has tested their models in independent populations. One important factor that makes researchers hesitate to do so is the concerns of small sample sizes and the heterogeneity of asthma exacerbations. We applied the RF models built with the Stage 1 samples to predict the independent Stage 2 samples (Figure 2). Overall, the independent test samples paralleled the predictive accuracy of the Stage 1 (training) samples with increasing numbers of SNPs until 160 SNPs. At 160, the replicating AUC reached its maximum and flattens out thereafter. As such, we cite the 160 SNP model as our best performing model. The independent replication AUCs are obviously higher than 0.5, indicating true predictability of the RF models. However, they are lower than the training and internal cross-validation AUCs for 160 and 320 SNPs, suggesting certain degree of over-fitting may still exist.

As discussed above, clinical traits alone did not produce desirable predictability for asthma exacerbation. We did, however, exclude one predictor that is a strong predictor of severe exacerbations - prior exacerbations [37]. The rationale for excluding this predictor was that we were interested in developing a predictive model based upon determinants of exacerbations; these determinants would by their nature include both prior and current exacerbations. Moreover, since we sought to determine genetic predictors of exacerbations, the inclusion of prior exacerbations would mitigate the strength of the genetic association in our analyses.

**Figure 4:**
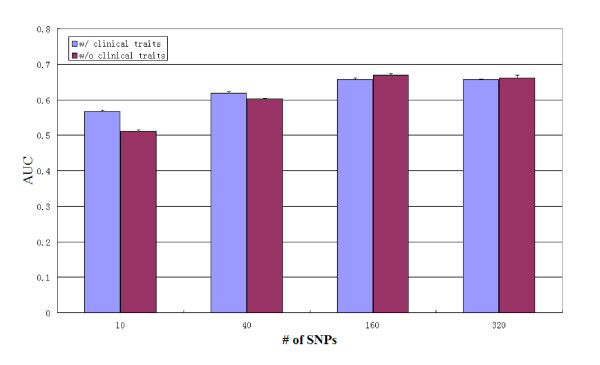
**Performance comparison of predicting severe asthma exacerbation with or without clinical traits**. Y-axis: AUC; X-axis: the number of SNPs used for prediction. Blue: SNPs plus clinical traits; Red: SNPs alone.

One of the reasons that clinical predictors may not have provided the same strength of prediction as our genetic models is that many of the clinical traits themselves are genetically determined. Indeed, our results (Figure 4) have shown that without clinical traits, SNPs alone can predict as well as with the clinical traits, suggesting asthma exacerbations are at least partly caused by genetic factors. For instance, among our clinical predictors, sex is genetically determined; age itself is not genetic, but it may be associated with age of onset due to the patient recruitment process, and age of onset in turn can be genetic [38,39]; and pre-bronchodilator FEV_1_% is influenced by genetics, especially in children.

There were several potential limitations to our study. We have already discussed the limitation due to a limited sample size. One potential problem is that with more than 160 SNPs, the training AUC keeps increasing (Figure 2), but the replicating AUC does not. This suggests that the chance of getting false positive SNPs increases with the number of SNPs used for prediction. One way to reduce false positive SNPs is to increase the sample size, which is costly.

## Conclusions

In conclusion, we have demonstrated that reasonable prediction of asthma exacerbations can be achieved through the use of hundreds of SNPs in a random forests model. This model can increase our understanding of the biologic mechanisms behind why only certain individuals with asthma are at risk for exacerbations, as well as the basis for the epistatic (gene-gene) interactions underlying asthma severity, providing insight into novel preventative and therapeutic strategies.

## Competing interests

The authors declare that they have no competing interests.

## Authors' contributions

MX carried out the experimental design, data analysis, interpretation and drafted the manuscript. KG directed the experimental design, data analysis, interpretation and participated in manuscript editing. AW and AL provided the phenotype information. JC participated in the experimental design. BEH imputed the missing values and participated in manuscript editing. AM provided the genotype files and participated in manuscript editing. STW had overall oversight of the study and helped prepare the manuscript. All authors read and approved the final manuscript.

## Pre-publication history

The pre-publication history for this paper can be accessed here:

http://www.biomedcentral.com/1471-2350/12/90/prepub

## Supplementary Material

Additional File 1 **Table S1**. Top 160 SNPs based on importance scores computed by RF. "GENE" is the closest gene to the SNP, "GENE REGION" is the relative location of the SNP to the gene. Click here for file
