# Supplementary material for: Genome Wide Association Study to predict severe asthma exacerbations in children using random forests classifiers
Source: BMC Med Genet. 2011 Jun 30;12:90. doi: 10.1186/1471-2350-12-90 (PMC3148549; doi:10.1186/1471-2350-12-90)
Supplement: Additional File 1 — Table S1. Top 160 SNPs based on importance scores computed by RF. "GENE" is the closest gene to the SNP, "GENE REGION" is the relative location of the SNP to the gene. [file 1471-2350-12-90-S1.DOC]

Table S1.

| RANK | SNP_ID | CLOSEST GENE | GENE REGION | IMPORTANCE |
| --- | --- | --- | --- | --- |
| 1 | rs9587342 | FAM155A | INTRON | 0.668984862 |
| 2 | rs6426881 | PBX1 | 3DOWN | 0.284006373 |
| 3 | rs1074119 | CARD11 | 5UP | 0.264645745 |
| 4 | rs858928 | NRXN1 | INTRON | 0.239529299 |
| 5 | rs12201938 | RREB1 | 5UP | 0.157168875 |
| 6 | rs4950949 | JARID1B | 5UP | 0.15058403 |
| 7 | rs8013206 | DNAL1 | INTRON | 0.142851267 |
| 8 | rs6795120 | FHIT | 3DOWN | 0.123859252 |
| 9 | rs4844324 | STARD8 | 5UP | 0.123272881 |
| 10 | rs13116794 | UNC5C | INTRON | 0.112146149 |
| 11 | rs1027486 | ATPBD4 | 5UP | 0.105369755 |
| 12 | rs2256966 | GRM6 | INTRON | 0.102630611 |
| 13 | rs4890712 | PIK3C3 | 5UP | 0.101186308 |
| 14 | rs8118279 | ZBP1 | INTRON | 0.093820495 |
| 15 | rs12632982 | TSC22D2 | 5UP | 0.091044071 |
| 16 | rs1474664 | KIAA1244 | 5UP | 0.090417944 |
| 17 | rs1575279 | RLN1 | 5UP | 0.081670034 |
| 18 | rs2015810 | MYT1 | INTRON | 0.080982255 |
| 19 | rs2580317 | CDYL2 | 5UP | 0.079098467 |
| 20 | rs7398015 | NUDT4 | 5UP | 0.078662771 |
| 21 | rs10938119 | EREG | 5UP | 0.076963806 |
| 22 | rs3891377 | CPEB2 | 5UP | 0.07341044 |
| 23 | rs6956055 | GTF2IRD1 | INTRON | 0.070568703 |
| 24 | rs8084688 | KLHL14 | 5UP | 0.06920131 |
| 25 | rs2327486 | SGK1 | 5UP | 0.068886541 |
| 26 | rs17588651 | CA10 | INTRON | 0.068370828 |
| 27 | rs2486405 | UST | INTRON | 0.06683245 |
| 28 | rs7742915 | BTBD9 | INTRON | 0.065168126 |
| 29 | rs556759 | CCS | INTRON | 0.058861155 |
| 30 | rs10777502 | NUDT4 | 5UP | 0.057418186 |
| 31 | rs3952257 | PTCHD1 | INTRON | 0.057083691 |
| 32 | rs9376317 | PERP | 3DOWN | 0.055850255 |
| 33 | rs868011 | SPEF2 | 5UP | 0.054449865 |
| 34 | rs4409685 | NEK7 | 5UP | 0.054343166 |
| 35 | rs932652 | HABP2 | INTRON | 0.054083452 |
| 36 | rs6474169 | ADAM18 | INTRON | 0.053116219 |
| 37 | rs1469243 | PDGFC | INTRON | 0.052127123 |
| 38 | rs11655341 | SLC39A11 | 3DOWN | 0.051995766 |
| 39 | rs2613217 | CDH17 | INTRON | 0.05082283 |
| 40 | rs6505069 | C17orf108 | 5UP | 0.050635554 |
| 41 | rs2270048 | LSM7 | INTRON | 0.050602908 |
| 42 | rs13064710 | THRB | 5UP | 0.050563278 |
| 43 | rs2886928 | HRASLS | 5UP | 0.047276321 |
| 44 | rs842647 | REL | INTRON | 0.045912493 |
| 45 | rs2868980 | SNN | 5UP | 0.0455201 |
| 46 | rs4925258 | CDH4 | INTRON | 0.045134669 |
| 47 | rs12557633 | REPS2 | 3UTR | 0.044408376 |
| 48 | rs1025493 | FBXL17 | INTRON | 0.044066132 |
| 49 | rs10504934 | CDH17 | INTRON | 0.043995253 |
| 50 | rs4770244 | FGF9 | 3DOWN | 0.04295735 |
| 51 | rs11160147 | PRIMA1 | 5UP | 0.042804259 |
| 52 | rs17078062 | FAM26D | 3DOWN | 0.041854328 |
| 53 | rs6937549 | T | 3DOWN | 0.041218709 |
| 54 | rs10786364 | AVPI1 | INTRON | 0.039765034 |
| 55 | rs7994213 | PCDH20 | 3DOWN | 0.039240232 |
| 56 | rs9539014 | PCDH20 | 3DOWN | 0.039144446 |
| 57 | rs1883181 | OFCC1 | 3DOWN | 0.039126822 |
| 58 | rs10046543 | GPR37 | 3DOWN | 0.03861262 |
| 59 | rs968061 | EREG | 5UP | 0.038598629 |
| 60 | rs6968538 | DKFZp564N2472 | 5UP | 0.038373241 |
| 61 | rs2235207 | TIPRL | INTRON | 0.038346456 |
| 62 | rs6563756 | COG6 | 3DOWN | 0.038296249 |
| 63 | rs7581039 | GYPC | 5UP | 0.037817902 |
| 64 | rs4845623 | IL6R | INTRON | 0.037457304 |
| 65 | rs169112 | NRG3 | 5UP | 0.037381074 |
| 66 | rs7028894 | CD274 | INTRON | 0.037132678 |
| 67 | rs6539726 | TMTC2 | 3DOWN | 0.037131311 |
| 68 | rs5919549 | OPHN1 | INTRON | 0.036497446 |
| 69 | rs6617168 | CHM | 3DOWN | 0.035775135 |
| 70 | rs378609 | KL | 5UP | 0.035743641 |
| 71 | rs2319453 | CA10 | INTRON | 0.035726079 |
| 72 | rs4975032 | UBE2K | 5UP | 0.035622058 |
| 73 | rs7632256 | SRGAP3 | INTRON | 0.035576384 |
| 74 | rs9389020 | TAAR1 | 3DOWN | 0.035439061 |
| 75 | rs732807 | FLJ44815 | 3DOWN | 0.035185192 |
| 76 | rs9520255 | FAM155A | 3DOWN | 0.035086914 |
| 77 | rs270443 | WWOX | 3DOWN | 0.034825783 |
| 78 | rs7815608 | MLZE | 3DOWN | 0.034457178 |
| 79 | rs737544 | MARCH3 | 5UP | 0.034198972 |
| 80 | rs17646697 | SULF1 | INTRON | 0.033908419 |
| 81 | rs6550227 | EFHB | 5UP | 0.033547744 |
| 82 | rs2014595 | TMEM35 | INTRON | 0.033438382 |
| 83 | rs1895301 | FOXI1 | 3DOWN | 0.033407979 |
| 84 | rs2063988 | EPHB6 | 5UP | 0.033322102 |
| 85 | rs10801929 | TTF2 | 5UP | 0.032855236 |
| 86 | rs16901238 | OSGIN2 | 5UP | 0.032761483 |
| 87 | rs11051128 | TSPAN11 | 5UP | 0.032525632 |
| 88 | rs10833325 | PRMT3 | INTRON | 0.03231946 |
| 89 | rs4684237 | C3orf19 | INTRON | 0.032197584 |
| 90 | rs1941580 | PIK3C3 | 5UP | 0.031836691 |
| 91 | rs12445719 | PRKCB1 | INTRON | 0.031444982 |
| 92 | rs1881959 | FAM19A2 | INTRON | 0.03102603 |
| 93 | rs2025582 | FAM20B | 5UP | 0.030697155 |
| 94 | rs2659695 | GATA2 | 5UP | 0.030545646 |
| 95 | rs10745641 | NUDT4 | 5UP | 0.029515357 |
| 96 | rs2206860 | DNAH8 | INTRON | 0.029494229 |
| 97 | rs6862273 | ADCY2 | 5UP | 0.029459742 |
| 98 | rs2046626 | TBC1D9 | INTRON | 0.029255093 |
| 99 | rs2512033 | MATN2 | INTRON | 0.028883171 |
| 100 | rs10496476 | DPP10 | INTRON | 0.028850932 |
| 101 | rs616246 | PFKFB3 | 5UP | 0.028848902 |
| 102 | rs12424252 | XRCC6BP1 | 3DOWN | 0.02878733 |
| 103 | rs2109818 | PID1 | INTRON | 0.02859706 |
| 104 | rs475738 | FGF20 | 3DOWN | 0.028540467 |
| 105 | rs6844368 | FRAS1 | INTRON | 0.028526977 |
| 106 | rs2277945 | FOXI1 | INTRON | 0.028011911 |
| 107 | rs12804250 | CADM1 | 3DOWN | 0.027902973 |
| 108 | rs318802 | ANKRD55 | INTRON | 0.027876459 |
| 109 | rs842639 | REL | 5UP | 0.027726674 |
| 110 | rs2197159 | PHB | 3DOWN | 0.027713601 |
| 111 | rs2223236 | OFCC1 | 3DOWN | 0.027616041 |
| 112 | rs2758555 | SFTPD | 3DOWN | 0.02743295 |
| 113 | rs742538 | BTBD9 | INTRON | 0.026931625 |
| 114 | rs1218427 | FRMD4A | INTRON | 0.026707115 |
| 115 | rs8065779 | SOX9 | 3DOWN | 0.026701038 |
| 116 | rs13254844 | NEFL | 5UP | 0.026551944 |
| 117 | rs6585810 | BTBD16 | INTRON | 0.026394948 |
| 118 | rs6682924 | BSDC1 | 5UP | 0.026393314 |
| 119 | rs11876215 | PIK3C3 | 5UP | 0.026384834 |
| 120 | rs1363097 | NUDT12 | 5UP | 0.026184286 |
| 121 | rs10843846 | TSPAN11 | 5UP | 0.026083948 |
| 122 | rs563530 | CD300E | 3DOWN | 0.026024407 |
| 123 | rs2724706 | DZIP1L | INTRON | 0.025983102 |
| 124 | rs10063386 | FBXL17 | INTRON | 0.025672727 |
| 125 | rs8008963 | FSCB | 3DOWN | 0.025651763 |
| 126 | rs2117516 | PTPRN2 | INTRON | 0.025476336 |
| 127 | rs10814983 | SLC1A1 | 5UP | 0.025410333 |
| 128 | rs1333922 | DBC1 | 5UP | 0.025376905 |
| 129 | rs1039115 | PIK3C3 | 5UP | 0.02525934 |
| 130 | rs10985334 | DAB2IP | INTRON | 0.025245583 |
| 131 | rs884206 | HLF | INTRON | 0.025228571 |
| 132 | rs872296 | PLEKHG3 | 5UP | 0.024972707 |
| 133 | rs2730091 | SH3GL3 | INTRON | 0.024943863 |
| 134 | rs4784744 | CETP | INTRON | 0.024823795 |
| 135 | rs1001990 | MKI67 | 5UP | 0.024809979 |
| 136 | rs5919577 | YIPF6 | 5UP | 0.024524873 |
| 137 | rs12629111 | MYRIP | INTRON | 0.023945089 |
| 138 | rs4793790 | MMD | 3DOWN | 0.023912949 |
| 139 | rs944093 | FARP1 | INTRON | 0.023586999 |
| 140 | rs761913 | CXorf27 | 3DOWN | 0.02349238 |
| 141 | rs12709230 | C16orf68 | 5UP | 0.023393492 |
| 142 | rs17039556 | FSTL5 | 3DOWN | 0.023202024 |
| 143 | rs10889026 | DAB1 | INTRON | 0.023174819 |
| 144 | rs1950610 | RPS29 | 3DOWN | 0.023131388 |
| 145 | rs6542033 | GYPC | 5UP | 0.023017259 |
| 146 | rs9314809 | ANXA1 | 3DOWN | 0.022836663 |
| 147 | rs225559 | TMEM132B | 3DOWN | 0.022656195 |
| 148 | rs35862 | IGSF11 | INTRON | 0.022610706 |
| 149 | rs8130408 | KCNJ6 | INTRON | 0.022560386 |
| 150 | rs12602671 | WSCD1 | 5UP | 0.022475583 |
| 151 | rs2461526 | NCOR2 | 5UP | 0.022401325 |
| 152 | rs10493474 | CTH | 3DOWN | 0.022307246 |
| 153 | rs6525226 | OPHN1 | INTRON | 0.02208203 |
| 154 | rs2137704 | PCDH9 | 5UP | 0.022069068 |
| 155 | rs12131981 | ST3GAL3 | INTRON | 0.021919964 |
| 156 | rs10934622 | SEMA5B | INTRON | 0.021683139 |
| 157 | rs1015990 | C16orf68 | 5UP | 0.021665695 |
| 158 | rs11896906 | TPO | INTRON | 0.021642803 |
| 159 | rs1026926 | UNC5C | INTRON | 0.021470597 |
| 160 | rs2395983 | SCG2 | 3DOWN | 0.021295041 |

Top 160 SNPs based on importance scores computed by RF. “GENE” is the closest gene to the SNP, “GENE REGION” is the relative location of the SNP to the gene.
